# Enhancing recruitment of individuals living with frailty, multimorbidity and cognitive impairment to Parkinson’s research: experiences from the PRIME-UK cross-sectional study

**DOI:** 10.1093/ageing/afae108

**Published:** 2024-05-23

**Authors:** Emma Tenison, Matthew D Smith, Danielle Pendry-Brazier, Anisha Cullen, Fiona E Lithander, Yoav Ben-Shlomo, Emily J Henderson

**Affiliations:** Ageing and Movement Research Group, Department of Population Health Sciences, Bristol Medical School, University of Bristol, Bristol BS8 1NU, UK; Older People’s Unit, Royal United Hospitals Bath NHS Foundation Trust, Bath BA1 3NG, UK; Ageing and Movement Research Group, Department of Population Health Sciences, Bristol Medical School, University of Bristol, Bristol BS8 1NU, UK; Older People’s Unit, Royal United Hospitals Bath NHS Foundation Trust, Bath BA1 3NG, UK; Ageing and Movement Research Group, Department of Population Health Sciences, Bristol Medical School, University of Bristol, Bristol BS8 1NU, UK; Ageing and Movement Research Group, Department of Population Health Sciences, Bristol Medical School, University of Bristol, Bristol BS8 1NU, UK; Ageing and Movement Research Group, Department of Population Health Sciences, Bristol Medical School, University of Bristol, Bristol BS8 1NU, UK; Liggins Institute, University of Auckland, Auckland 1023, New Zealand; Ageing and Movement Research Group, Department of Population Health Sciences, Bristol Medical School, University of Bristol, Bristol BS8 1NU, UK; The National Institute for Health and Care Research Applied Research Collaboration West (NIHR ARC West) at University Hospitals Bristol and Weston NHS Foundation Trust, Bristol, UK; Ageing and Movement Research Group, Department of Population Health Sciences, Bristol Medical School, University of Bristol, Bristol BS8 1NU, UK; Older People’s Unit, Royal United Hospitals Bath NHS Foundation Trust, Bath BA1 3NG, UK

**Keywords:** Parkinson’s disease, mental capacity, inclusivity, underserved populations, older people

## Abstract

**Background and objectives:**

People with parkinsonism who are older, living in a care home, with frailty, multimorbidity or impaired capacity to consent are under-represented in research, limiting its generalisability. We aimed to evaluate more inclusive recruitment strategies.

**Methods:**

From one UK centre, we invited people with parkinsonism to participate in a cross-sectional study. Postal invitations were followed by telephone reminders and additional support to facilitate participation. Personal consultees provided information on the views regarding research participation of adults with impaired capacity. These approaches were evaluated: (i) using external data from the Parkinson’s Real World Impact assesSMent (PRISM) study and Clinical Practice Research Datalink (CPRD), a sample of all cases in UK primary care, and (ii) comparing those recruited with or without intensive engagement.

**Results:**

We approached 1,032 eligible patients, of whom 542 (53%) consented and 477 (46%) returned questionnaires. The gender ratio in PRIME-UK (65% male) closely matched CPRD (61% male), unlike in the PRISM sample (46%). Mean age of PRIME participants was 75.9 (SD 8.5) years, compared to 75.3 (9.5) and 65.4 (8.9) years for CPRD and PRISM, respectively. More intensive engagement enhanced recruitment of women (13.3%; 95% CI 3.8, 22.9%; *P* = 0.005), care home residents (6.2%; 1.1, 11.2%; *P* = 0.004), patients diagnosed with atypical parkinsonism (13.7%; 5.4, 19.9%; *P* < 0.001), and those with a higher frailty score (mean score 0.2, 0.1, 0.2; *P* < 0.001).

**Conclusions:**

These recruitment strategies resulted in a less biased and more representative sample, with greater inclusion of older people with more complex parkinsonism.

## Key Points

- Parkinson’s research often under-represents older adults, those living with frailty, cognitive impairment and/or multimorbidity.
- The strategies used in the PRIME-UK cross-sectional study supported the recruitment of a more representative sample.
- The empirical evidence that follow-up telephone calls supported participation is important for ethics committees to consider.
- The questionnaire data collected will better reflect real-world clinical practice.

## Introduction

Parkinson’s disease (PD) is uncommon before the age of 50 years, and prevalence rises with age [1]. Therefore, many people with PD are also living with frailty, a syndrome of loss of physiological reserve resulting in increased vulnerability to adverse health outcomes [2], and/or multimorbidity, which describes the co-existence of two or more long-term conditions [3]. Cognitive impairment is a common non-motor symptom of PD, with a mean point prevalence of dementia amongst PD patients of 24.5% (95% CI 17.4; 31.5%) [4], and a further 25.8% of those with PD without dementia are estimated to have mild cognitive impairment [5].

It is widely recognised that research populations need to reflect the population being treated in clinical practice, yet populations recruited into clinical research are often unrepresentative of the real-world population [6]. Many observational studies of people with parkinsonism frequently exclude patients based on age, comorbidities, cognitive impairment or the inability to consent, limiting the generalisability of the findings. Exclusion of patients unable to consent may be the decision of the researchers, as well as ethics committees stipulating that a study should only be conducted with patients able to provide informed consent [7].

In addition to the explicit exclusion of PD patients from research within eligibility criteria, for example, due to age [8, 9], participants can be implicitly excluded due to participation being associated with physical, financial or logistical burden [10] or because study information is not provided in an accessible format, for example, for someone with a visual impairment. A review of consecutive published randomised controlled trials (RCTs) over an 18-month period, albeit not specific to PD, highlighted that on average, three potential participants needed to be screened for each participant included due to high exclusion and refusal rates [11]. Retention can also be challenging; in a meta-analysis of individual participant-level data, an increasing number of comorbidities was associated with increased trial attrition [12], which contributes to a reluctance to include people with multimorbidity in research studies. To ensure findings are generalisable and reflect real-world experience, it remains important not to exclude on the basis of comorbidities but to factor the likely increased attrition into sample size calculations [12].

Under-representation of adults lacking capacity to consent in both PD and other disease areas is multi-factorial and not solely due to restrictive eligibility criteria [13]. Shepherd has described three main categories of barriers to the inclusion of adults lacking capacity to consent: methodological issues, such as a lack of appropriate outcome measures for populations with impaired capacity; structurally determined factors, such as the resource-intensive nature of research in this group and a lack of resources to support proxy decision-makers; and systemic factors, including navigating complex legal frameworks and ethical processes [13]. Furthermore, paternalistic attitudes can suggest adults lacking the capacity to consent must be protected from research [13]. It is vital that we overcome the challenges and barriers to inclusion to both ensure that research is representative and recognise that research participation is a positive experience for people with PD and their caregivers [14].

We designed an inclusive study and embedded processes to tackle the barriers to participation for under-served groups with parkinsonism. In addition, we sought to evaluate the success of these approaches by (i) comparing the characteristics of recruited participants with external data on participants in the Parkinson’s Real World Impact assesSMent (PRISM) study, a European cross-sectional study [15], and patients with prevalent parkinsonism in the Clinical Practice Research Datalink (CPRD), a United Kingdom (UK) primary care database, which should capture all diagnosed cases. We (ii) used internal data on those recruited with or without intensive engagement.

Our hypothesis was that the sample of patients recruited to the PRIME cross-sectional study would more closely match the characteristics of patients in the CPRD database (‘gold standard’) and would be more representative than those in the PRISM survey.

## Methods

### Study design and population

This was a single-centre, cross-sectional, questionnaire study of people with parkinsonism living in the catchment area of Royal United Hospital Bath NHS Foundation Trust, a UK district general hospital, together with their primary informal caregiver, if appropriate. The protocol has been reported previously [16] but is summarised briefly below, and further details are provided in supplemental methods.

### Recruitment procedures

Eligible participants were identified from lists of patients coded with parkinsonism during inpatient admission and lists of patients seen in movement disorder outpatient clinics.

Potential participants who did not respond to the invitation letter received one or more telephone calls from the study team to answer questions about study participation, ascertain how the team could support participation, identify if there were any requirements for translation and, where necessary, assess their capacity to consent to taking part. The call numbers were logged.

### Adults lacking capacity to consent to participation in the study

In accordance with the Mental Capacity Act (2005), which covers England and Wales [17], patients were assumed to have capacity to consent to the study unless there was evidence to suggest otherwise. In some cases, a capacity assessment was triggered. If the potential participant was identified as not having capacity, a personal consultee, usually a close family member or friend (who was not a paid carer), was sought to review the study requirements and offer advice about the wishes and views of the patient on taking part in research at the time they had capacity.

If the consultee advised that the person would have consented at the time they had capacity, they signed the consultee declaration form. The personal consultee, or another close friend/relative of the person with parkinsonism, was asked to complete questionnaires on their behalf. Where no personal consultee was available, the patient was excluded.

### Comparison to other populations

The characteristics of participants recruited to this cross-sectional study were compared to the characteristics of patients in CPRD GOLD [18], a UK primary care database, registered at the midpoint of 2019 and with one or more clinical codes for parkinsonism prior to this. As CPRD is a routine healthcare rather than a research database, this sample includes all diagnosed subjects with parkinsonism under contributing primary care practices and can be considered the ‘gold standard’ as it is rare for patients to opt out of the database.

Another comparator sample, the PRISM study, was selected as it was also cross-sectional in design [15]. This European study recruited people with PD with assistance from advocacy groups in each country and asked them to self-complete an online survey [15]. The UK sub-component of this dataset was used for this comparison to avoid cross-country differences in research participation.

### Statistical analysis

We compared the following characteristics of patients recruited with and without one or more telephone calls: age, gender, parkinsonism duration, care home status, type of parkinsonism (atypical versus idiopathic PD), frailty score (using the SHARE-FI75+ tool [19]), non-motor symptom burden (using the Non-Motor Symptom Questionnaire (NMSQ) [20]) and patient activation level, a measure of self-management capability (using the Patient Activation Measure [21]).

Means were compared using a *t*-test and proportions using a *z*-test of proportions, in which the null hypothesis was that there was no difference in means or proportions between groups [22]. Stata 17 was used for data cleaning and analysis.

## Results

### Recruitment flowchart

**Figure 1:**
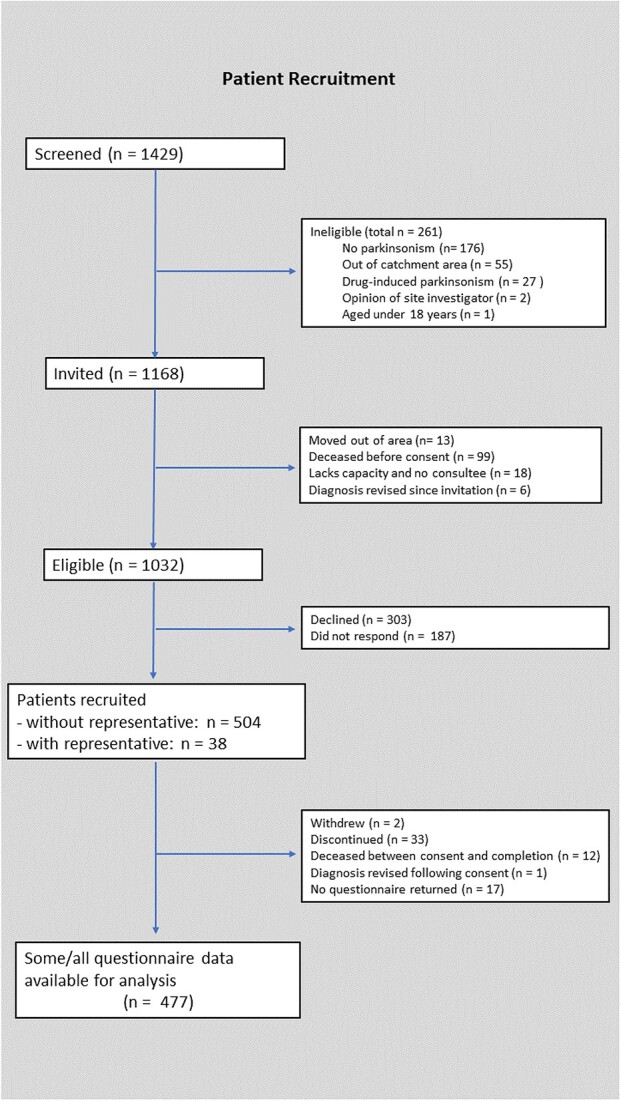
Flowchart of patient recruitment.

1,429 patients were screened for inclusion in the study, 1,168 of whom met criteria for invitation (Figure 1). 136 of those originally invited were subsequently found to be ineligible because they had moved out of the area (*n* = 13), were deceased before consenting to the study (*n* = 99), their diagnosis of parkinsonism had been revised to an alternative diagnosis (*n* = 6) or they lacked capacity to consent and had no personal consultee (*n* = 18). Of 1,032 patients confirmed as eligible, 542 (53%) participants were recruited, including 38 who lacked capacity to consent and so took part with a representative. 477 patient participants returned at least some completed questionnaire responses.

### Strategies to enhance recruitment

Of the 1,168 invited patients, 490 did not require a telephone call because they consented, declined or were found to be deceased before a follow-up call was made. 650 out of 678 (95.9%) individuals requiring a call were reached on one or more occasions (including contact with their formal/informal caregiver), and a total of over 900 calls were made overall, with patients receiving between 1 and 6 calls (weighted average 1.37 calls), as shown in Table 1. Over 300 follow-up invitation packs were sent out after the initial invitation to patients who had not received or had mislaid the initial pack, with some patients being sent up to three packs.

**Table 1:** Number of successful telephone calls, in which a patient or their formal/informal caregiver was reached, conducted to 678 individuals requiring telephone contact

Total number of successful calls	Number of patients receiving each number of calls
0	28
1	438
2	157
3	47
4	6
5	1
6	1

Of the 650 patients who received one or more telephone reminders following a postal invitation, 167 subsequently consented to the study, which represents 30.8% of the 542 recruited patient participants. 267 who received one or more telephone prompts declined to take part, 158 did not provide a final decision and 58 were subsequently determined to be ineligible. 22 out of 38 (57.9%) participants recruited with a representative were recruited following one or more calls.

Table 2 shows the total number of calls received by patients who were recruited following one or more telephone calls; although the yield reduced with each subsequent call, a small number of participants were recruited after three or four calls. 156 out of 167 (93.4%) participants recruited following a call were recruited after one or two calls, with a further 11 out of 167 (6.6%) recruited after three or four calls.

**Table 2:** The number of calls received prior to recruitment in 167 patients recruited after a telephone prompt

	Number of calls prior to recruitment			
	1	2	3	4
Patient recruited without a representative (*n* = 145 patients)	94	42	9	0
Patient recruited with a representative (*n* = 22 patients)	19	1	1	1

### Comparison to other study populations

Table 3 shows the characteristics of participants who took part in the PRIME-UK cross-sectional study, alongside UK participants in the PRISM study and individuals under follow-up within CPRD at the midpoint of 2019. PRISM appears to have oversampled women relative to PRIME (19.0% difference, 95% CI 11.0; 27.0, *P* < 0.001), while the gender ratio in PRIME more closely matches CPRD (4.2% difference, 95% CI -0.3; 8.7, *P* = 0.07). The mean age of PRIME participants was 75.9 (SD 8.5) years, while it was 75.3 (9.5) years (difference in mean age 0.6 years, 95% CI -0.3; 1.1, *P* = 0.18) and 65.4 (8.9) years (difference in mean age 10.5 years, 95% CI 9.1; 11.1, *P* < 0.001) for CPRD patients and PRISM participants, respectively. More than half of PRIME participants were 75 years and over, in comparison to PRISM, in which 16% of participants were ≥75 years. This more closely matches the age of patients within the CPRD database, of which 53% were ≥75 years.

**Table 3:** Comparison of PRIME participants to other study populations (CPRD and UK participants of the PRISM cross-sectional study)

Characteristics	PRIME *n* (%)	PRISM (UK) [15] *n* (%)	CPRD^*^n (%)
Gender			
Male	310 (65.0)	97 (46.0)	3,719 (60.8)
Female	167 (35.0)	112 (53.1)	2,397 (39.2)
Other	0 (0)	1 (0.5)	0 (0)
Prefer not to say	0 (0)	1 (0.5)	0 (0)
Age			
Mean (SD)	75.9 (8.5)	65.4 (8.9)	75.3 (9.5)
Age group			
<40	0 (0)	0 (0)	6 (0.1)
40–59	21 (4.4)	60 (28.2)	382 (6.3)
60–64	29 (6.1)	35 (16.4)	530 (8.7)
65–69	59 (12.4)	41 (19.3)	762 (12.5)
70–74	102 (21.4)	41 (19.3)	1,204 (19.7)
75–79	113 (23.7)	24 (11.3)	995 (16.3)
80–84	79 (16.6)	9 (4.2)	1,391 (22.7)
85–89	62 (13.0)	1 (0.5)	648 (10.6)
90+	12 (2.5)	0 (0)	198 (3.2)
Missing	0 (0)	2 (0.9)	0 (0)
Ethnicity^†^			
White	471 (98.7)	Not recorded	1,059 (93.9)
Asian/Asian British: Chinese	1 (0.2)		2 (0.2)
Asian/Asian British: Indian	0 (0)		15 (1.3)
Asian/Asian British: Pakistani	0 (0)		5 (0.4)
Any other Asian background	0 (0)		8 (0.7)
Black/African/Caribbean/Black British: African	0 (0)		1 (0.1)
Black/African/Caribbean/Black British: Caribbean	0 (0)		11 (1.0)
Any other ethnic group	4 (0.8)		9 (0.8)
Mixed	0 (0)		4 (0.4)
Missing/not recorded	1 (0.2)		†
Diagnosis			
Parkinson’s disease	412 (86.4)	Recruited people with ‘PD’	5123 (83.7)
Parkinson’s disease dementia	10 (2.1)		213 (3.5)
Dementia with Lewy Bodies	17 (3.6)		351 (5.7)
Progressive supranuclear palsy	12 (2.5)		88 (1.4)
Multiple system atrophy	6 (1.3)		42 (0.7)
Vascular parkinsonism	10 (2.1)		266 (4.4)
Corticobasal degeneration	0 (0)		25 (0.4)
Don’t know	10 (2.1)		11 (0.2)
Disease duration (years)			
Median (range)	5 (2–9)	6 (0–42)	3.5 (0–37.5)
<2 years	79 (16.6)	33 (15.5)	1932 (31.6)
2–5 years	142 (29.8)	60 (28.2)	1988 (32.5)
5–10 years	154 (32.3)	71 (33.3)	1681 (27.5)
10–20 years	88 (18.5)	40 (18.8)	463 (7.6)
20+ years	14 (2.9)	4 (18.9)	55 (0.9)

^*^ Patients under CPRD follow-up on the mid-point of 2019.

^†^Ethnicity available for *n* = 1,114 of patients under CPRD follow-up on the midpoint of 2019.

PRISM restricted recruitment to people with PD, so comparison of the type of diagnosis is not possible. Comparison of PRIME with CPRD data suggests that PRIME may have slightly under-represented people with PD dementia (2.1% in PRIME versus 3.5% in CPRD, 95% CI for difference 0.0; 2.8), as well as people with atypical parkinsonism (total 9.5% in PRIME diagnosed with DLB, PSP, MSA, CBD and vascular parkinsonism, versus 11.7% in CPRD, 95% CI for difference −5.0; 0.6), although there is only weak evidence for this (*P* = 0.10 and *P* = 0.15, respectively). PRIME participants were also less ethnically diverse than patients for whom ethnicity was recorded within CPRD, which was unsurprising given the ethnic distribution of the population in the PRIME-UK catchment area compared to CPRD, which is much more geographically heterogeneous.

### The characteristics of patient participants recruited with or without telephone reminder

While there was little difference in mean age and disease duration between those recruited with (*n* = 142) or without (*n* = 335) a reminder call, there were other marked differences (Table 4). Participants recruited after one or more telephone calls were more likely to be female, living in a care home, diagnosed with an atypical form of parkinsonism, and to have a higher SHARE-FI75+ frailty score. There was moderate evidence to support a slightly higher non-motor symptom burden and lower patient activation score, measures that were only captured in those with capacity to consent, amongst those recruited after one or more calls.

**Table 4:** The characteristics of patient participants recruited with or without telephone reminder

Patient characteristics	No telephone call required (total *n* = 335)	Consented after 1 or more telephone calls (total *n* = 142)	Difference between groups: one or more calls versus no calls (95% CI)	*P*-value
Mean (SD) age (years)	75.4 (8.4)	76.8 (8.7)	1.4 (−0.3; 3.1)	0.10
Gender (*n*/% male)	231 (69.0)	79 (55.6)	−13.3 (−3.8; −22.9)	0.005
Mean (SD) PD duration (years)	6.4	6.1	−0.3 (−1.4; 0.8)	0.58
Care home status (*n*/% in a care home)	10 (3.0)	13 (9.2)	6.2 (1.1; 11.2)	0.004
Type of parkinsonism (*n*/% with atypical PD)	26 (7.8)	29 (20.4)	13.7 (5.4; 19.9)	<0.001
Mean (SD) frailty (SHARE-FI 75+) score	0.4 (0.3)	0.6 (0.3)	0.2 (0.1; 0.2)	<0.001
Mean (SD) non-motor symptom burden (NMSQ)^*^	10.4 (5.2)	11.8 (5.9)	1.4 (0.2; 2.7)	0.02
Mean (SD) patient activation score (PAM)^*^	54.9 (13.4)	51.1 (15.3)	−1.50 (−0.9; −6.8)	0.01

^*^ Only assessed in patients with capacity to consent to the study.

## Discussion

We have evaluated and found evidence that the additional supportive methods used in the PRIME-UK cross-sectional study resulted in a more representative sample of patients with parkinsonism. The mean age of PRIME participants (76 years) was similar to the mean age of patients in the CPRD database (75 years) and higher than for UK participants in the PRISM cross-sectional study (65 years), as well as many other observational studies, including the Non-motor International Longitudinal Study (66 years) [23] and the PRIAMO multicentre study (67 years) [24]. Although we did not randomise potential participants to different recruitment strategies, the finding that individuals recruited after one or more telephone reminders had a specific phenotype provides empirical evidence to support this recruitment strategy.

A Cochrane systematic review of strategies to improve recruitment, albeit to trials rather than observational studies, concluded that telephone reminders to non-responders following postal invitation improved recruitment by 6% (95% CI 3%–9%) [25], which supports our finding. This review did not focus specifically on recruiting hard-to-reach groups, such as older adults. A systematic review of strategies used within RCTs and observational studies to improve recruitment and/or retention of adults aged 65 and over found that hand delivery of questionnaires by somebody known to the participant improved survey response [26]. We adapted this, ensuring that the initial invitation letter came from each patient’s named specialist rather than from an unfamiliar healthcare or research professional.

A strength of this study is that it was designed with the aim of being as inclusive as possible. These approaches have led to the recruitment of a population that better reflects the ‘real world’. We acknowledge that the study region under-represents ethnic minorities and hence is not representative of the UK overall. The PRIME-UK cross-sectional study population also included patients with a diagnosis other than idiopathic PD, and the choice of PRISM as a comparison study population is limited by this study excluding diagnoses other than PD. While the comparison with CPRD diagnoses suggests that PRIME may have slightly under-represented those with PD dementia and atypical parkinsonian syndromes, our study relied on self-reported diagnosis, which may not accurately reflect the working diagnosis. Additionally, the 3.5% coded as PD dementia within CPRD may also be an under-estimate since dementia is believed to be under-captured in electronic records [27].

We successfully recruited 23 patients (4.8%) living in either a residential or nursing home. Care home residents may still have been under-represented in this study, since a UK survey has previously reported that 4% of over 65-year-olds and 15% of those aged 85 years and over live in care homes [28]. A study of 135 people with idiopathic PD in North-East England found that 19 (14.1%) were living in a care home [29]. We successfully recruited 38 adults with impaired capacity to consent, following the involvement of a personal consultee. An ongoing ‘Study Within a Trial’ (SWAT), randomly allocating proxy decision-makers to receive standard study information with or without a newly designed decision aid, will add to the evidence base on how best to recruit this under-served group [30].

There were challenges reaching and identifying consultees for adults with impaired capacity to consent. The recently published NIHR INCLUDE Impaired Capacity to Consent Framework [31] offers practical recommendations to improve this process, along with the ‘CONSULT’ programme, which is exploring a decision support intervention for consultees [32]. There is an increasing effort to encourage individuals to consider their wishes for taking part in research in case their capacity to make decisions becomes impaired in the future [33]. Furthermore, the CONSULT-ADVANCE workstream is exploring the views of patients, researchers and healthcare professionals around advance research planning [34].

While our more representative sample makes these findings more generalisable to the UK population with parkinsonism, we recognise that there may still be non-response bias in our sample, from patients who did not consent to the study and from those that ceased enrollment. Our detailed study flowchart provides crucial information to allow readers to assess the generalisability of the findings and recognises that many studies fail to describe the population from which the study sample was drawn.

The success of the strategies used in this study has important implications for improving the inclusivity of future studies. Despite this being an observational study with no in-person visits, the resources required to deliver the study, including over 900 reminder telephone calls after a postal invitation (estimated to equate to over 150 hours of person time, assuming an average call duration of 10 minutes), were considerable. Our data highlight the positive impact these calls had in terms of both the overall response rate and enabling us to reach people with atypical parkinsonian syndrome, care home residents and individuals living with frailty. Calls made to support questionnaire completion after consent required additional resources but facilitated the participation of individuals who may otherwise have declined. This is relevant to researchers designing a study aiming to be inclusive, as it can inform the planning of resources and staff needed and help justify additional costs when applying for funding. It also provides powerful empirical evidence that can be used when applying to ethics committees, who need to consider the recruitment burden on potential participants, that follow-up telephone calls can be justified as a strategy to support the enrolment of hard-to-reach individuals. Additionally, it helps reduce the research inequities that often exist with less supportive recruitment methods, which will inevitably bias the inclusion of more affluent, educated and able participants [35, 36].

This cross-sectional study has successfully enabled a broader group of people with parkinsonism to take part in research and has added evidence around strategies that can support diversity and inclusion. The more representative sample of patients recruited means that the collected data better reflects the heterogeneity of patients seen in clinical practice. Failure to include such patients will misleadingly result in under-estimating the true burden and costs of providing care to the whole parkinsonism population. Applying these strategies within the setting of PD clinical trials could help to generate evidence relevant to the broader range of patients under the care of movement disorders services. Future research should identify the most cost-effective recruitment strategies given limited funding, though funders should be encouraged to ‘ring-fence’ clinical research funding specifically to enhance equity of access to research opportunities.

## Supplementary Material

aa-23-1960-File002_afae108
