# Supplementary material for: Enhancing recruitment of individuals living with frailty, multimorbidity and cognitive impairment to Parkinson’s research: experiences from the PRIME-UK cross-sectional study
Source: Age Ageing. 2024 May 23;53(5):afae108. doi: 10.1093/ageing/afae108 (PMC11116826; doi:10.1093/ageing/afae108)
Supplement: aa-23-1960-File002_afae108 [file aa-23-1960-file002_afae108.docx]

# Enhancing recruitment of individuals living with frailty, multimorbidity and cognitive impairment to Parkinson’s research: experiences from the PRIME-UK cross-sectional study

# Supplemental methods

The study protocol has been published previously [1] but some sections are reproduced below for reference.

## Eligibility criteria

Inclusion criteria

- Have a diagnosis of parkinsonism (including idiopathic Parkinson’s disease, progressive supranuclear palsy, corticobasal degeneration, multiple system atrophy, dementia with Lewy Bodies, vascular parkinsonism), made by a movement disorder specialist (a physician sub-specialising in neurology or geriatric medicine)
- Be willing to participate
- Have the ability to provide informed consent to participate or, where unable to do so due to cognitive impairment, availability of a close friend or relative to act as a personal consultee
- Be aged 18 years or over
- Live in the catchment area of Royal United Hospitals Bath NHS Foundation Trust (RUH Bath)

Exclusion criteria

- Individuals with drug-induced parkinsonism
- Individuals who lack capacity to consent to participate but do not have anyone who can be a consultee to provide advice regarding their wishes and views
- Current medical, cognitive or psychosocial issue or co-enrolment in another study that, in the opinion of the site investigator, would interfere with adherence to study requirements (e.g. individuals in the last days/weeks of life)

## Recruitment procedures: capacity assessment and identification of consultees

Patients were recruited to the study over a 16-month period from September 2020.

In accordance with the Mental Capacity Act 2005 [2], patients were assumed to have capacity to consent to the study unless there was evidence to suggest otherwise. Situations which prompted capacity assessment included return of incomplete or partially completed consent forms; an individual (such as care home staff or a family member), who answered the phone on behalf of a patient during a follow-up call, expressing concern that the patient may struggle to understand the study information. If a capacity assessment was triggered, this was conducted by telephone by a trained member of the team, who was typically a clinician. This was performed in accordance with the Mental Capacity Act 2005 two-stage test [2] and documented on the RUH Bath capacity proforma. Steps were taken to optimise the setting to facilitate the potential participant being able to make a capacitous decision (e.g. by calling back on another occasion; by ensuring that a family member or friend was with the potential participant during the assessment, if possible).

Where it was established that an individual did not have capacity to make this decision, personal consultees were identified from next of kin details held within clinical records, discussion with care home staff and, where relevant, asking to speak to anyone who lived with or supported the potential patient participant. Nominated consultees, who are individuals such as a general practitioner, solicitor or member of care home staff, who are not connected with the study, were not used in place of personal consultees. This was for two main reasons: firstly, such individuals acting in a professional capacity were deemed less likely to be aware of the patient’s prior wishes and views relevant to the decision; secondly, an individual requiring a consultee also required an individual who was sufficiently close to them to act as their representative, a role which a nominated consultee would likely not be able to fulfil.

## Ethics and consent

This protocol was approved by the London- Brighton & Sussex Research Ethics Committee (REC) on 27 July 2020; REC reference 20/LO/0890.

All participants provided written informed consent or, in the case of patients who lacked capacity to consent to study participation, a consultee provided advice on their prior wishes and signed a consultee declaration if they believed they would have consented at a time they had capacity.

## Data collection

Recruited participants completed a single questionnaire booklet at home during the study period. If a patient participant lacked capacity to consent to the study, a representative (who was often the person who acted as consultee) completed a specially designed patient questionnaire booklet on their behalf. The questionnaire booklets included questions about demographics, medical and medication history, as well as validated tools to assess a variety of metrics including frailty, nutritional risk, sarcopenia, wellbeing. The full patient questionnaire booklet additionally contained questionnaires to assess overall non-motor symptom burden and autonomic symptoms, whilst the booklet for representatives assessed neuropsychiatric symptoms in further detail.

Where participants had capacity but had a physical inability to mark responses on the questionnaire (e.g. due to visual impairment, tremor or bradykinesia), assistance with marking a physical response could be undertaken by another person, which could include their paid carer, with the answer communicated by the participant. In some cases, a member of the research team assisted them to complete questionnaires fully or partly over the telephone and, where needed, this was done over the course of multiple phone calls to minimise burden/fatigue. Where relevant, we liaised with care home staff to ask them to assist with physical completion.

## References

1. Tenison E, Lithander FE, Smith MD, Pendry-Brazier D, Ben-Shlomo Y, Henderson EJ. Needs of patients with parkinsonism and their caregivers: a protocol for the PRIME-UK cross-sectional study. BMJ Open. 2022;12(5):e057947

2. Mental Capacity Act 2005- Code of Practice 2007 [cited 12th February 2024]. Available from: <https://www.legislation.gov.uk/ukpga/2005/9/pdfs/ukpgacop_20050009_en.pdf>.
